# Supplementary material for: Characterisation, identification, clustering, and classification of disease
Source: Sci Rep. 2021 Mar 8;11:5405. doi: 10.1038/s41598-021-84860-z (PMC7940639; doi:10.1038/s41598-021-84860-z)
Supplement: Supplementary file 1 — Supplementary Information. [file 41598_2021_84860_MOESM1_ESM.pdf]

Characterisation, identification, clustering, and classification of disease - Supplementary Material

A.J. Webster, K. Gaitskell, I. Turnbull, B.J. Cairns, R. Clarke

February 1, 2021

Selection of diseases for study

Diseases were considered at the 3- and 4-digit ICD-10 code levels, and either amalgamated into a single group, split into 4-digit codes, or excluded at either the 3-digit or 4-digit level. The criteria for exclusion of diseases are described in the first section of the main article. In the rare occasions where splitting and re-grouping 4-digit codes required a new 3-digit code, then an “a” was appended to the 3-digit code, for example see S22 and H11 in table 1. Any 3- or 4-digit diseases that were studied are listed in table 1, along with any 4-digit exclusions from the same 3-digit category. In addition, each disease was assigned a category of Acute (1), Chronic(2), Infectious (I), Injury (Inj), or Excluded (E), to reflect either the nature of the disease or whether it was excluded.

Statistical tests - covariance matrices

Meaningful statistical tests and clustering requires the statistical model and its estimated confidence intervals (covariance matrix), to be good approximations. For sufficiently large sample sizes n, the maximum likelihood estimates (mles) will be normally distributed [1]. Unfortunately there is no general rule to determine how large is “sufficiently large” [1], and this is influenced by the number of cases in each category of a categorical variable. Nonetheless we dropped all diseases with less than 50 cases, because with up to 12 risk factors, some with three categories, it seemed unreasonable to expect a meaningful fit. We subsequently excluded diseases if any of their parameters or covariance matrices were undefined, and ensured that the estimated covariance matrices are positive definite, which we tested by checking that all the eigenvalues are positive. We also excluded diseases if their covariance matrices' eigenvalues were unusually large, indicating either a poorly defined covariance or excessively large confidence intervals for one or more parameter. Provided the number of cases in each categorical variable are large enough, then the estimated covariance matrices and their eigenvalues will be proportional to $1/n$ [1]. Plotting for each disease the logarithm of n times the maximum eigenvalue of the estimated covariance matrice versus $\log(n)$ confirmed that $\hat{\Sigma} \propto 1/n$ in most cases (figure 1), and a histogram of the maximum eigenvalues indicated a small number of outliers (figure 1), that indicate very large confidence intervals for one or more parameters. Therefore as a simple criteria to exclude diseases with poorly estimated parameters or poorly defined covariance matrices, we excluded diseases if the maximum eigenvalue was more than 2.5 standard deviations larger than the mean (calculated with outliers). Such diseases would be expected to either not be statistically significant, or to not allow meaningful statistical tests due to poorly estimated covariances. Note that female diseases tend to have larger eigenvalues due to more

parameters being estimated for women than men (HRT and parity are adjusted for in women only).

We separately considered the 400 most common first diseases in an ICD-10 chapter that occurred in the study period from when a participant joined the study until 31st March 2017. Following the clinical selection of diseases and tests of the covariance matrices the number of diseases was reduced to 343 diseases in men and 346 in women. Of these, 150 diseases in men and 140 in women were statistically significant at the 0.05 level after a Bonferoni multiple-testing adjustment, as discussed in the main text (see table 1 in the main text). The proportional hazards model was tested and diseases rejected if they failed the test at the 0.05 level after an FDR multiple-testing adjustment [2], leaving 138 diseases in men and 127 diseases in women. Of the remaining diseases, 86 were present in both men and women. These were used in the clustering and identification studies.

Sensitivity analyses

As described in Section 5 of the main article, sensitivity analyses were run that clustered diseases after refitting the proportional hazards model with additional exclusions of data, and a model that used tertiles in place of a linear fit to continuous parameters.

Considering firstly sensitivity to exclusions - Taking the 24 clusters of diseases from figure 5 of the main article, the data were refit for each disease, excluding any disease that was present in the same cluster. This was to prevent the possibility of clusters being influenced by data from the same individual, through diseases from different ICD-10 chapters that affect the same person. The clustering is almost identical to the original analysis (figure 2), consistent with the quantitative comparisons in the main text.

To test the sensitivity of clusterings to the model being used to fit the data, we refit a proportional hazards model to the same set of diseases but with sex-specific tertiles for height, BMI, SBP, and year of birth. We continued to stratify by year of birth and adjust by the age at which participants enter the study, and similarly to Section 3, before clustering we used marginalisation to compare a baseline of non-smokers, non-diabetic, rarely drink, and minimum tertiles for height, BMI, and SBP, to parameters for regularly smoking, diabetes, regularly drinking, and maximum tertiles for height, BMI, and SBP. The resulting clusters are similar (figure 3), consistent with the quantitative comparisons in the main text. The model with tertiles continued to find diseases with statistically significant differences between men and women. The diseases with statistically significant differences between men and women (at the 0.05 level after an FDR adjustment), were also similar to those in the main article (figure 4). Only kidney cancer, pulmonary embolisms, and atrial fibrillation had statistically significant differences in the analysis with tertiles, and were not present in the original analysis. Overall there were 4 diseases with different associations that were common to both analyses, and without BMI as a risk factor, kidney stones remained common to the results of both analyses (figure 4).

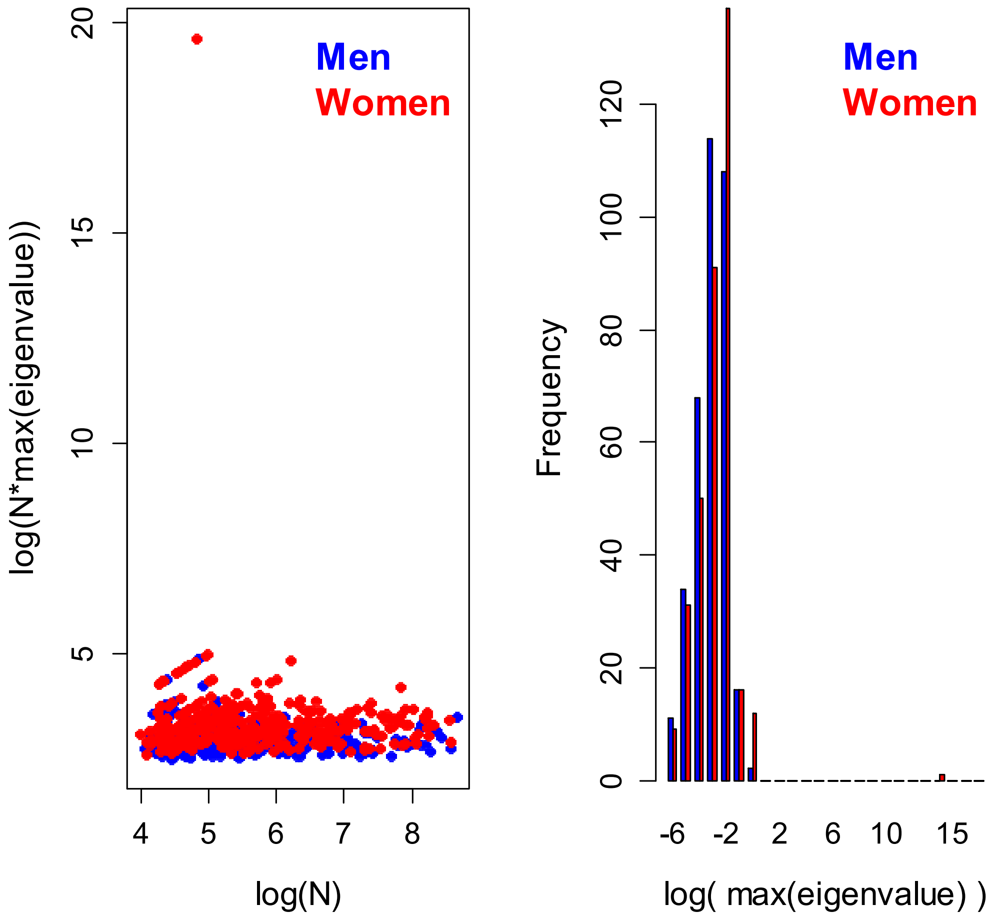

Figure 1: Covariance matrices can be characterised by their eigenvalues, the largest of which will be proportional to $1/N$ where N is the number of cases. Outliers indicate unreliable estimates for the covariance matrix typically caused by too few data in one or more category being estimated, such as diabetes for example. Diseases with obvious outliers are excluded from further analysis because reliable tests and confidence intervals require a reliable covariance matrix.

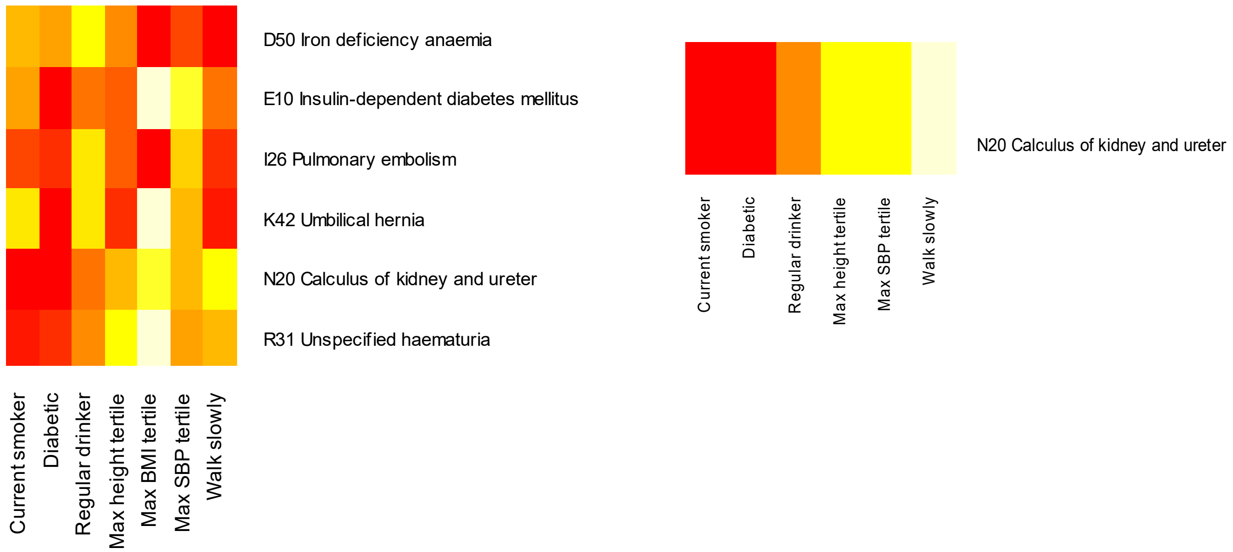

Figure 2: Data with exclusions for prior disease from the same clusters as in figure 5 of the main article, were used to refit the model, to test the sensitivity of clustering to prior disease from the same individual (but different ICD-10 chapter). The results are almost identical to figure 5 of the main article, in agreement with quantitative comparisons of differences in the clusterings. Labels are coloured by their first ICD-10 digit and appended with their cluster number. The dendrogram is coloured with the top 22 clusters in the dendrogram. Associations with potential risk factors are indicated by the heat map, with red for increased risk, white reduced risk, and orange neutral. The figure was produced with R [3] and package “gplots” [4].

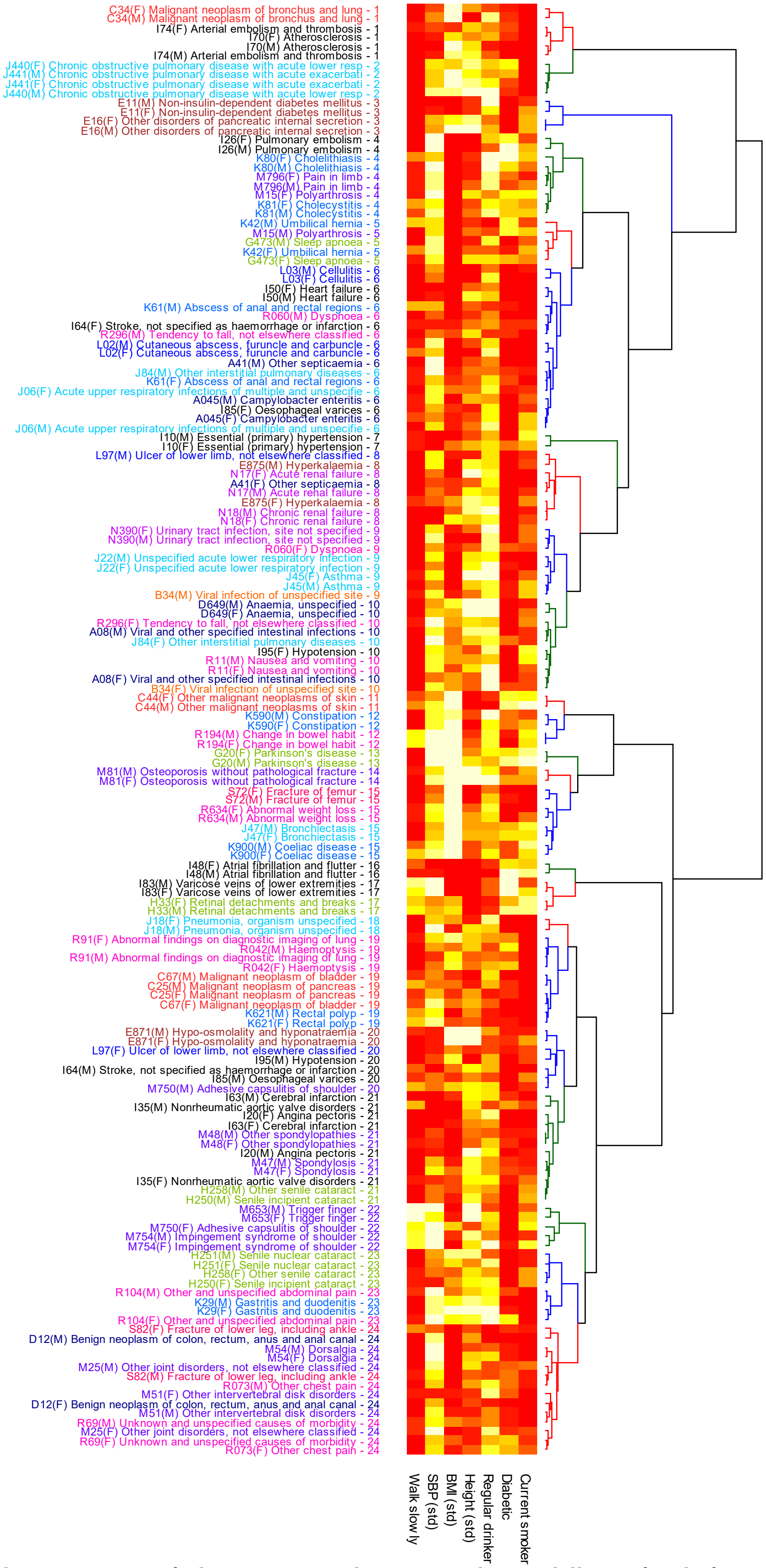

Figure 3: To test the sensitivity of clusterings to changes in the modelling of risk factors, continuous measures for height, BMI, and SBP, were replaced with sex-specific tertiles for height, BMI, SBP, and also year of birth (previously tertiles of men and women together). Despite differences to the model, clustering is similar to figure 5 of the main article, consistent with quantitative comparisons. As for figure 2, labels are coloured by their first ICD-10 digit, and the heat map has red for increased risk, white reduced risk, and orange neutral. The figure was produced with R [3] using packages "dendextend" [5] and "gplots" [4].

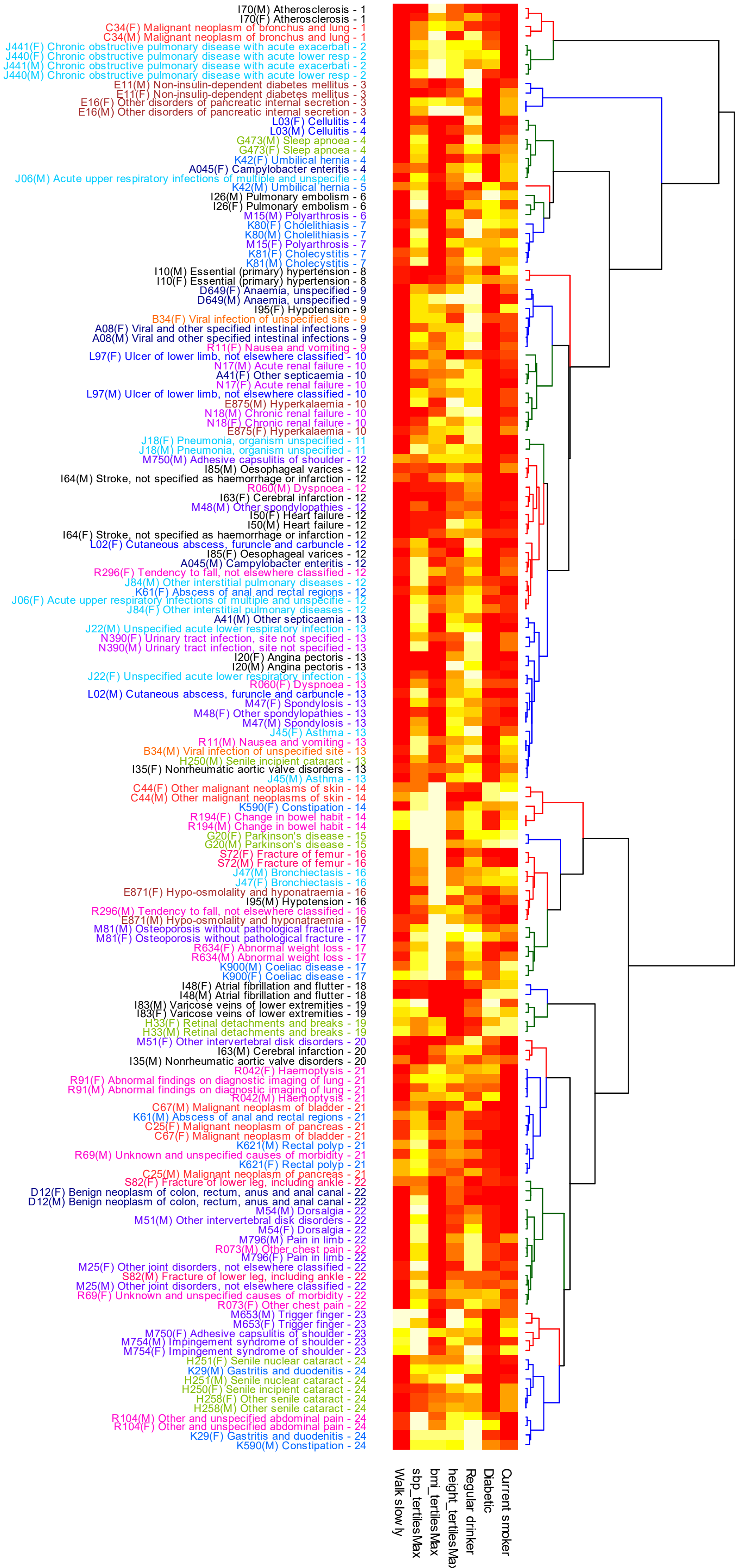

Figure 4: The same diseases in men and women can have statistically significant differences in their associations with risk factors at the 0.05 level after an FDR multiple-testing adjustment, here shown for a model with sex-specific tertiles for height, BMI, SBP, and year of birth. With all potential risk factors (left), and without BMI (right). Associations with greater risk for women than men are indicated by red, lower risks are white, orange is neutral. The figure was produced with R [3] using packages "dendextend" [5] and "gplots" [4].

Table 1: Diseases were selected on criteria described in the first section of the main article, and assigned a category of Acute (1), Chronic(2), Infectious (I), Injury (Inj), or Excluded (E). “ICDs in group”, are 4-digit ICD-10 coded diseases included in the group labelled “Code”, “Excluded ICDs” are any ICDs excluded from the 3- or 4-digit ICD-10 coded group. Diseases in “Code” appended with “*” are included in the clustering and classification of studies, an “a” was appended when an extra disease code was required . Where the number of cases is given in brackets, they were not among the 400 most common diseases for that gender, and not studied. The table was produced using R and package “xtable” [6].

Code	Category	N (male)	N (female)	ICDs in group	Excluded ICDs
A045*	I	122	90	A045	
A047	I	69	89	A047	
A048	I	130	240	A048	
A08*	I	274	365	A080, A081, A082, A083, A084, A085	
A09	I	1875	3078	A09, A090, A099	
A41*	I	1264	1210	A410, A411, A412, A413, A414, A415, A418, A419	
B02	I	80	106	B020, B021, B022, B023, B027, B028, B029	
B07	I	158	207	B07	
B34*	I	297	437	B340, B341, B342, B348, B349	
B37	I	177	205	B370, B371, B372, B373, B374, B377, B378, B379	
C15	1	370	119	C150, C151, C152, C153, C154, C155, C158, C159	
C16	1	222	85	C160, C161, C162, C163, C164, C165, C166, C168, C169	
C18	1	965	756	C180, C181, C182, C183, C184, C185, C186, C187, C188, C189	
C19	1	133	(69)	C19	
C20	1	453	254	C20	
C22	1	151	97	C220, C221, C227, C229	
C25*	1	248	217	C250, C251, C252, C253, C254, C257, C258, C259	
C32	1	89	(4)	C320, C321, C322, C323, C329	
C34*	1	840	737	C340, C341, C342, C343, C348, C349	
C43	1	552	556	C430, C431, C432, C433, C434, C435, C436, C437, C439	
C44*	1	4510	3676	C440, C441, C442, C443, C444, C445, C446, C447, C448, C449	
C45	1	182	(40)	C450, C451, C457, C459	
C49	1	79	(51)	C490, C491, C492, C493, C494, C495, C496, C498, C499	
C50	1	(32)	5130	C500, C501, C502, C503, C504, C505, C506, C508, C509	
C54	1	(0)	698	C540, C541, C542, C543, C548, C549	
C56	1	(0)	427	C56	
C61	1	4200	(0)	C61	
C64	1	367	205	C64	
C67*	1	868	260	C670, C671, C672, C673, C674, C675, C676, C678, C679	
C71	1	236	143	C710, C711, C712, C713, C714, C715, C716, C717, C718, C719	
C73	1	(46)	124	C73	
C786	E	(36)	80		C786
C787	E	97	83		C787

C795	E	114	79		C795
C833	1	154	124	C833	
C851	1	72	(42)	C851	
C859	1	91	93	C859	
C90	1	220	182	C900, C901, C902, C903	
C911	1	132	(63)	C911	
D03	1	193	216	D030, D031, D032, D033, D034, D035, D036, D037, D038	
D04	1	137	141	D040, D041, D042, D043, D044, D045, D046, D047, D049	
D05	1	(3)	872	D050, D051, D057, D059	
D06	1	(0)	74	D060, D069	
D075	1	190	(0)	D075	
D10	1	100	104	D100, D101, D102, D103, D104, D105, D106, D109	
D11	1	115	132	D110, D117, D119	
D12*	1	5260	3741	D120, D121, D122, D123, D124, D125, D126, D127, D128, D129	
D17	1	848	872	D170, D171, D172, D173, D174, D175, D176, D177, D179	
D180	1	213	260	D180	
D21	1	79	109	D210, D211, D212, D213, D214, D215, D216, D219	
D22	2	514	763	D220, D221, D222, D223, D224, D225, D226, D227, D229	
D23	1	583	648	D230, D231, D232, D233, D234, D235, D236, D237, D239	
D24	1	(3)	362	D24	
D25	1	(0)	1909	D250, D251, D252, D259	
D27	1	(0)	555	D27	
D32	1	(51)	137	D320, D321, D329	
D34	1	(18)	91	D34	
D351	1	(48)	184	D351	
D352	1	92	(62)	D352	
D361	1	79	98	D361	
D374	1	71	(73)	D374	
D414	1	201	75	D414	
D45	1	85	(42)	D45	
D46	2	102	(54)	D460, D461, D462, D464, D465, D466, D467, D469	
D50	1	1884	2774	D500, D501, D508, D509	
D619	1	85	(47)	D619	
D630	E	(64)	95		D630
D649*	1	1844	2068	D649	
D696	1	126	(67)	D696	
D70	1	333	567	D70	
D751	1	110	(42)	D751	
D86	1	73	77	D860, D861, D862, D863, D868, D869	
E04	2	153	606	E040, E041, E042, E048, E049	
E05	1	(45)	203	E050, E051, E052, E053, E055, E058, E059	
E10	1	99	99	E101, E102, E103, E104, E105, E106, E108, E109	

E11*	2	450	212	E110, E111, E112, E113, E114, E115, E116, E119	
E16*	1	182	112	E161, E162, E168, E169	E160
E21	1	(64)	262	E210, E211, E212, E213, E214, E215	
E66	2	142	351	E660, E662, E668, E669	
E831	2	291	168	E831	
E835	2	151	187	E835	
E86	1	166	145	E86	
E871*	1	171	248	E871	
E875*	1	204	112	E875	
E876	1	(52)	83	E876	
F05	1	140	98	F050, F051, F058, F059	
F100	E	138	(58)		F100
F103	E	125	(47)		F103
F32	1	186	203	F320, F321, F322, F323, F328, F329	
F41	1	145	275	F410, F411, F412, F418, F419	
F45	1	(58)	89	F450, F452, F453, F454, F458, F459	
G122	1	101	79	G122	
G20*	2	208	101	G20	
G30	2	78	(69)	G300, G301, G308, G309	
G35	1	(50)	124	G35	
G40	1	284	215	G400, G401, G402, G403, G404, G405, G406, G407, G408, G409	
G43	1	211	670	G430, G431, G432, G433, G438, G439	
G442	1	(54)	116	G442	
G45	2	937	755	G450, G451, G453, G454, G458, G459	
G473*	2	816	356	G473	
G479	E	170	114		G479
G50	1	(48)	97	G500, G501, G508, G509	
G510	1	141	126	G510	
G551	2	72	97	G551	
G560	1	1740	3331	G560	
G562	1	328	212	G562	
G576	1	130	603	G576	
G61	1	86	(46)	G610, G618, G619	
G62	E	157	102	G628, G629	G620, G621, G622
G81	1	86	91	G811, G819	
H00	1	393	337	H000, H001	
H020	2	281	191	H020	
H021	2	192	86	H021	
H023	2	121	237	H023	
H024	2	242	391	H024	
H028	E	367	415		H028
H029	E	150	197		H029

H042	2	107	238	H042	
H045	2	175	434	H045	
H11a	1	85	116	H111, H112, H113, H114, H118, H119	
H250*	2	626	861	H250	
H251*	2	1750	2533	H251	
H258*	2	694	943	H258	
H259	2	387	610	H259	
H264	2	73	92	H264	
H268	2	168	234	H268	
H269	2	4214	5757	H269	
H33*	2	925	692	H330, H331, H332, H333, H334, H335	
H34	2	148	120	H341, H342, H348, H349	
H353	2	391	685	H353	
H358	E	141	114		H358
H40	2	659	825	H400, H401, H402, H403, H404, H405, H408, H409	
H431	1	89	(65)	H431	
H50	1	137	206	H500, H501, H502, H503, H504, H505, H506, H508, H509	
H53	1	226	230	H530, H531, H532, H533, H534, H538, H539	
H610	1	134	117	H610	
H65	1	227	230	H650, H651, H652, H653, H654, H659	
H66	I	83	93	H660, H661, H662, H663, H664, H669	
H72	1	143	147	H720, H721, H722, H728, H729	
H811	2	91	115	H811	
H830	1	189	239	H830	
H919	2	81	110	H919	
I10*	2	209	372	I10	
I20*	1	1237	1055	I200, I201, I208, I209	
I21	1	2626	967	I210, I211, I212, I213, I214, I219	
I24	1	165	111	I240, I241, I248, I249	
I251	2	3812	1569	I251	
I259	2	252	238	I259	
I26*	1	938	873	I260, I269	
I319	1	94	(44)	I319	
I34	2	162	107	I340, I341, I342, I349	
I35*	2	330	243	I350, I351, I352, I358, I359	
I44	1	356	202	I440, I441, I442, I443, I444, I446, I447	
I45	1	98	(61)	I451, I452, I453, I454, I455, I456, I458, I459	
I46	1	84	(47)	I460, I461, I469	
I471	1	270	369	I471	
I472	1	84	(43)	I472	
I48*	1	2541	1642	I48, I480, I481, I482, I483, I484, I489	
I495	1	80	(58)	I495	

I50*	2	348	235	I500, I501, I509	
I60	1	119	232	I600, I601, I602, I603, I604, I605, I606, I607, I608, I609	
I61	1	234	171	I610, I611, I612, I613, I614, I615, I616, I618, I619	
I62	1	127	(59)	I620, I621, I629	
I63*	1	1294	864	I630, I631, I632, I633, I634, I635, I636, I638, I639	
I64*	1	180	126	I64	
I65	2	117	(69)	I650, I651, I652, I653	
I70*	2	167	96	I700, I701, I702, I708, I709	
I71	2	344	(65)	I710, I711, I712, I713, I714, I715, I716, I719	
I739	2	109	(61)	I739	
I74*	1	137	78	I740, I741, I742, I743, I744, I745, I748, I749	
I771	1	115	(63)	I771	
I80	1	787	607	I800, I801, I802, I803, I808, I809	
I83*	1	1042	1718	I830, I831, I832, I839	
I84	1	3491	3935	I840, I841, I842, I843, I844, I845, I846, I847, I848, I849	
I85*	1	153	84	I850, I859	
I95*	E	276	222	I950, I951, I958, I959	I952
J02	I	118	157	J020, J028, J029	
J03	I	69	123	J030, J038, J039	
J06*	I	123	153	J060, J068, J069	
J10	I	71	81	J100, J101, J108	
J13	I	(51)	78	J13	
J18*	I	2828	2395	J180, J181, J182, J188, J189	
J22*	I	1451	1389	J22	
J31	2	80	(64)	J310, J311, J312	
J32	2	280	285	J320, J321, J322, J323, J324, J328, J329	
J33	1	660	318	J330, J338, J339	
J342	1	637	380	J342	
J343	1	86	74	J343	
J348	E	262	262		J348
J35	2	106	162	J350, J351, J352, J353, J358, J359	
J36	I	103	(61)	J36	
J383	E	185	102		J383
J387	E	75	100		J387
J392	I	102	80	J392	
J40	1	(46)	85	J40	
J440*	2	393	319	J440	
J441*	2	240	191	J441	
J449	2	75	(46)	J449	
J45*	1	281	585	J450, J459	
J47*	2	82	163	J47	
J69	E	151	88		J690, J691, J698

J84*	2	198	141	J840, J841, J848, J849	
J90	1	619	506	J90	
J93	1	107	(67)	J930, J931, J938, J939	
J969	E	78	(54)		J969
J984	E	126	137		J984
K011	1	165	209	K011	
K02	2	632	685	K020, K021, K022, K028, K029	
K045	1	(32)	75	K045	
K047	I	132	170	K047	
K05	1	90	158	K050, K051, K052, K053, K054, K055, K056	
K083	1	330	361	K083	
K088	E	74	106		K088
K130	1	130	165	K130	
K132	1	81	76	K132	
K137	E	339	467		K137
K148	E	133	155		K148
K20	1	914	968	K20	
K21	2	1853	2628	K210, K219	
K221	1	497	336	K221	
K222	1	237	172	K222	
K227	2	468	259	K227	
K228	E	(58)	94		K228
K25	1	308	432	K250, K251, K253, K254, K255, K256, K257, K259	
K26	1	252	141	K260, K261, K262, K263, K264, K265, K266, K267, K269	
K29*	1	2576	3948	K29, K290, K291, K293, K294, K295, K296, K297, K298	K292
K30	2	1124	2346	K30	
K317	1	283	722	K317	
K318	E	78	119		K318
K35	I	468	523	K350, K351, K352, K353, K358, K359	
K37	I	(53)	80	K37	
K40	2	6071	426	K400, K401, K402, K403, K404, K409	
K41	2	(29)	152	K410, K412, K413, K414, K419	
K42*	2	883	276	K420, K421, K429	
K43	2	326	452	K43, K430, K431, K433, K435, K436, K439	
K44	2	1112	2138	K440, K449	
K50	1	78	122	K500, K501, K508, K509	
K51	1	250	231	K510, K511, K512, K513, K514, K515, K518, K519	
K528	E	(50)	141		K528
K529	1	1168	1919	K529	
K55	2	84	112	K550, K551, K552, K558, K559	
K565	1	86	146	K565	
K566	E	163	221		K566

K57	2	3144	4356	K570, K571, K572, K573, K574, K575, K578, K579	
K58	1	(50)	155	K580, K589	
K590*	1	652	1173	K590	
K60	1	319	185	K600, K601, K602, K603, K604, K605	
K61*	I	214	94	K610, K611, K612, K613, K614	
K620	1	126	111	K620	
K621*	1	1013	775	K621	
K623	1	(40)	170	K623	
K625	1	1213	1395	K625	
K627	E	86	(11)		K627
K628	E	218	252		K628
K635	1	2807	2352	K635	
K638	E	110	157		K638
K64	1	337	356	K640, K641, K642, K643, K644, K645, K648, K649	
K70	E	100	(24)		K700, K701, K702, K703, K704, K709
K80*	2	1172	2863	K800, K801, K802, K803, K804, K805, K808	
K81*	1	259	351	K810, K811, K818, K819	
K831	1	115	100	K831	
K851	1	(66)	92	K851	
K859	1	85	76	K859	
K85a	E	100	78		K85
K900*	1	108	173	K900	
K920	1	166	133	K920	
K921	1	302	175	K921	
K922	1	1219	1308	K922	
L02*	I	626	511	L020, L021, L022, L023, L024, L028, L029	
L03*	I	1827	1449	L030, L031, L032, L033, L038, L039	
L05	I	98	(34)	L050, L059	
L08	I	159	126	L080, L088, L089	
L309	E	(68)	81		L309
L40	2	96	138	L400, L401, L403, L404, L405, L408, L409	
L439	1	80	150	L439	
L57	2	703	755	L570, L573, L574, L575, L578, L579	
L60	2	177	203	L600, L601, L602, L603, L608, L609	
L720	1	432	475	L720	
L721	1	754	814	L721	
L729	1	180	228	L729	
L81	1	154	228	L810, L812, L813, L814, L816, L817, L818, L819	
L82	2	733	802	L82	
L85	2	191	191	L851, L852, L858, L859	
L900	1	(15)	96	L900	
L905	1	319	521	L905	

L918	E	81	(70)		L918
L97*	2	141	81	L97	
L980	1	80	78	L980	
L988	E	130	168		L988
L989	E	991	1072		L989
M06	2	112	325	M060, M061, M062, M063, M064, M068, M069	
M10	2	194	(29)	M100, M109	
M13	2	199	320	M130, M131, M138, M139	
M15*	2	279	430	M150, M151, M152, M153, M154, M158, M159	
M16	2	2688	3825	M160, M161, M162, M163, M164, M165, M166, M167, M169	
M17	2	2941	3322	M170, M171, M172, M173, M174, M175, M179	
M18	2	110	406	M180, M181, M183, M189	
M19	2	718	1203	M190, M191, M192, M198, M199	
M201	2	246	2647	M201	
M202	2	133	373	M202	
M204	2	71	254	M204	
M205	2	(60)	237	M205	
M23	2	3515	3130	M230, M231, M232, M233, M234, M235, M236, M238, M239	
M241	E	71	105		M241
M25*	2	1148	1649	M250, M251, M252, M253, M254, M255, M256, M257, M258, M259	
M316	E	(60)	125		M316
M43	2	102	181	M430, M431, M432, M435, M436, M438	
M47*	2	331	435	M471, M472, M478, M479	
M48*	2	502	487	M480, M481, M484, M485, M488, M489	
M50	2	195	181	M500, M501, M502, M503, M508, M509	
M51*	2	927	1121	M510, M511, M512, M513, M514, M518, M519	
M533	E	(14)	81		M533
M54*	2	1460	2121	M540, M541, M542, M543, M544, M545, M546, M548, M549	
M653*	2	452	624	M653	
M654	1	(17)	77	M654	
M659	E	76	113		M659
M674	1	391	680	M674	
M70	E	253	204	M700, M701, M702, M703, M704, M705, M706, M707	M708, M709
M720	2	1497	488	M720	
M750*	2	187	376	M750	
M751	2	834	675	M751	
M753	2	(22)	79	M753	
M754*	2	899	1069	M754	
M758	E	101	103		M758
M771	1	74	83	M771	
M791	1	(67)	97	M791	
M796*	1	683	932	M796	

M798	E	778	726		M798
M809	2	(32)	94	M809	
M81*	2	100	690	M810, M815, M816, M818, M819	M814
M840	E	(36)	107		M840
M841	E	123	143		M841
M86	I	108	(43)	M861, M862, M864, M866, M868, M869	
M899	E	(62)	99		M899
N02	1	93	75	N020, N021, N022, N023, N028, N029	
N12	1	102	300	N12	
N13	1	556	303	N130, N131, N132, N133, N134, N135, N136, N137, N138, N139	
N17*	1	655	334	N170, N171, N178, N179	
N18*	2	198	88	N180, N181, N182, N183, N184, N185, N188, N189	
N20	1	1114	447	N200, N201, N202, N209	
N21	1	193	(29)	N210, N211, N218	
N23	1	241	106	N23	
N302	E	78	81		N302
N308	E	(67)	83		N308
N309	I	127	160	N309	
N318	E	(53)	82		N318
N320	1	381	(7)	N320	
N323	1	80	(17)	N323	
N328	E	768	395		N328
N329	E	127	(57)		N329
N35	1	541	367	N358, N359	
N390*	I	1776	1761	N390	
N393	E	(58)	1094		N393
N394	E	79	167		N394
N398	E	74	(32)		N398
N399	E	92	(38)		N399
N40	2	3353	(1)	N40	
N41	I	489	(0)	N410, N411, N412, N413, N418, N419	
N428	E	183	(0)		N428
N429	E	73	(0)		N429
N43	1	587	(0)	N432, N433, N434	
N459	I	218	(0)	N459	
N47	1	849	(0)	N47	
N480	1	121	(0)	N480	
N481	1	97	(0)	N481	
N486	1	203	(0)	N486	
N488	E	112	(0)		N488
N501	1	174	(0)	N501	
N508	E	591	(0)		N508

N60	1	(1)	295	N600, N601, N602, N603, N604, N608, N609	
N61	1	(4)	98	N61	
N62	1	(22)	88	N62	
N63	1	(8)	191	N63	
N645	E	(2)	113		N645
N648	E	(1)	81		N648
N80	2	(0)	162	N800, N801, N803, N804, N805, N806, N808, N809	
N81	2	(0)	3817	N810, N811, N812, N813, N814, N815, N816, N818, N819	
N832	E	(0)	610		N832
N84	1	(0)	2686	N840, N841, N842, N843, N848, N849	
N850	1	(0)	117	N850	
N858	E	(0)	226		N858
N87	1	(0)	156	N870, N871, N872, N879	
N898	E	(0)	110		N898
N904	1	(0)	106	N904	
N907	1	(0)	125	N907	
N908	E	(0)	132		N908
N92	2	(2)	1585	N920, N921, N923, N924, N925, N926	
N93	1	(0)	345	N930, N938, N939	
N95	2	(0)	1767	N950, N951, N952, N953, N958, N959	
N993	E	(0)	120		N993
R000	R	(54)	75	R000	
R001	R	202	76	R001	
R002	R	288	523	R002	
R040	R	452	323	R040	
R042*	R	304	233	R042	
R05	R	235	330	R05	
R060*	R	655	723	R060	
R065	R	203	104	R065	
R068	R	108	(67)	R068	
R072	R	862	935	R072	
R073*	R	1323	1395	R073	
R074	R	3581	3480	R074	
R101	R	977	1660	R101	
R102	R	(29)	108	R102	
R103	R	673	1251	R103	
R104*	R	1404	2261	R104	
R11*	R	356	706	R11	
R12	R	116	238	R12	
R13	R	860	1244	R13	
R14	R	(67)	130	R14	
R15	R	79	413	R15	

R17	R	96	(57)	R17
R18	R	104	144	R18
R190	R	(58)	170	R190
R194*	R	1560	3001	R194
R195	R	273	438	R195
R198	R	150	248	R198
R20	R	196	204	R200, R201, R202, R203, R208
R21	R	81	130	R21
R22	R	244	268	R220, R221, R222, R223, R224, R227, R229
R268	R	70	(73)	R268
R296*	R	114	109	R296
R298	R	176	164	R298
R31	R	2835	2358	R31
R32	R	77	136	R32
R33	R	1133	164	R33
R35	R	335	187	R35
R39	R	645	345	R391, R398
R410	R	200	172	R410
R42	R	449	419	R42
R490	R	69	(65)	R490
R50	R	332	355	R500, R501, R508, R509
R51	R	673	1181	R51
R53	R	190	243	R53
R55	R	1596	1255	R55
R568	R	297	218	R568
R59	R	228	232	R590, R591, R599
R600	R	(60)	74	R600
R634*	R	275	380	R634
R69*	R	941	1025	R69
R79	R	1686	146	R790, R798, R799
R876	R	(0)	128	R876
R91*	R	361	355	R91
R933	R	199	245	R933
R945	R	107	147	R945
S000	Inj	71	(68)	S000
S008	Inj	137	141	S008
S009	Inj	90	103	S009
S010	Inj	163	125	S010
S018	Inj	218	153	S018
S02	Inj	303	290	S020, S021, S022, S023, S024, S025, S026, S027, S028, S029
S065	Inj	144	94	S065
S066	Inj	79	(62)	S066

S099	Inj	262	241	S099
S224	Inj	166	(64)	S224
S22a	Inj	109	87	S220, S221
S270	Inj	75	(33)	S270
S32	Inj	253	377	S320, S321, S322, S323, S324, S325, S327, S328
S42	Inj	516	776	S420, S421, S422, S423, S424, S427, S428, S429
S430	Inj	102	(72)	S430
S52	Inj	602	2906	S520, S521, S522, S523, S524, S525, S526, S527, S528
S610	Inj	301	224	S610
S611	Inj	152	81	S611
S618	Inj	103	82	S618
S62	Inj	567	413	S620, S621, S622, S623, S624, S625, S626, S627, S628
S66	Inj	255	108	S660, S661, S662, S663, S664, S665, S666, S667, S668, S669
S681	Inj	100	(33)	S681
S72*	Inj	557	1040	S720, S721, S722, S723, S724, S727, S728, S729
S761	Inj	180	(18)	S761
S82*	Inj	773	1803	S820, S821, S822, S823, S824, S825, S826, S827, S828, S829
S860	Inj	167	(55)	S860
S92	Inj	169	182	S920, S921, S922, S923, S924, S925, S927, S929

References

[1] Hardle, W. & Simar, L. *Applied Multivariate Statistical Analysis* (Springer, 2015).

[2] Wasserman, L. *All of Statistics* (Springer, 2005).

[3] R Core Team. *R: A Language and Environment for Statistical Computing.* R Foundation for Statistical Computing, Vienna, Austria (2020).

[4] Warnes, G. R. *et al. gplots: Various R Programming Tools for Plotting Data* (2020).

[5] Galili, T. dendextend: an r package for visualizing, adjusting, and comparing trees of hierarchical clustering. *Bioinformatics* (2015).

[6] Dahl, D. B., Scott, D., Roosen, C., Magnusson, A. & Swinton, J. *xtable: Export Tables to LaTeX or HTML* (2019).
